# Investigating targeted and untargeted approaches to biomarker discovery in fearful, adult dogs

**DOI:** 10.1093/jas/skag260

**Published:** 2026-08-19

**Authors:** Scarlett R Burron, Candace C Croney, Dave J Seymour, Rachel Strassburger, Stephanie Cottee, Alexandra Harlander, Anna K Shoveller

**Affiliations:** Department of Animal Biosciences, University of Guelph, Guelph, ON N1G 2W1, Canada; Department of Comparative Pathobiology, Purdue University College of Veterinary Medicine, West Lafayette, IN 47907, United States; Research & Development, Trouw Nutrition, Boxmeer, NB 5830 AE, The Netherlands; Department of Animal Biosciences, University of Guelph, Guelph, ON N1G 2W1, Canada; Probiotech International, Saint-Hyacinthe, QC J2S 8L2, Canada; Department of Animal Biosciences, University of Guelph, Guelph, ON N1G 2W1, Canada; Department of Animal Biosciences, University of Guelph, Guelph, ON N1G 2W1, Canada

**Keywords:** canine behavior, tryptophan, metabolomics, fearfulness, biomarker discovery

## Abstract

Chronic fear and anxiety experienced by domestic dogs pose significant welfare concerns, yet the molecular mechanisms related to these emotional states remain poorly understood. The objective of this study was to explore physiological correlates of fearfulness in dogs using targeted and untargeted metabolomic approaches to identify metabolic markers. One hundred privately owned adult dogs of various breeds (48 females; 52 males, 4.2 ± 2.1 yr, and 25.7 ± 11.6 kg [mean ± SD]) were recruited for this study, spanning a range of owner-reported fearfulness categories/levels. Behavior was assessed using a previously published survey and the Canine Behavioral Assessment and Research Questionnaire (C-BARQ), resulting in 10 behavioral outcome categories. Serum samples were collected for enzyme-linked immunosorbent assay (ELISA) analysis of serotonin (5-HT) and 5-hydroxyindoleacetic acid (5-HIAA), alongside untargeted LC-MS metabolomics analysis. Ordinal logistic regression models assessed associations between metabolites and fear-related behavior categories, adjusting for age, sex, body weight, and dog as a random effect. Multiple testing for metabolomics data was controlled by Benjamini–Hochberg FDR (*P*_FDR_ < 0.05). Pathway analysis identified tryptophan metabolism enriched for touch sensitivity (*P*_FDR_ = 0.001) and pyrimidine metabolism enriched for dog-directed fear (*P*_FDR_ = 0.041). At the metabolite level, formiminoglutamic acid, a marker of impaired one-carbon metabolism, was positively associated with combined fear (C-BARQ), while lauric acid, a medium-chain fatty acid, was positively associated with touch sensitivity (*P*_FDR_ < 0.05). In targeted analyses, the 5-HIAA:5-HT ratio was inversely related to separation-related behavior (*P* = 0.03), and 5-HIAA was or tended to be weakly associated with combined fear (Temesi *P* = 0.04; C-BARQ *P* = 0.08), whereas 5-HT alone was not associated with any behavioral category. Metabolomics and ELISA rank estimates of 5-HT and 5-HIAA did not correlate, highlighting methodological differences and supporting the need to use both targeted and untargeted methods in biomarker discovery. Taken together, these exploratory results point to biologically plausible pathway-level patterns, particularly involving tryptophan, one-carbon/folate, and lipid metabolism. These may guide future research on nutritional and therapeutic strategies for fear-related behaviors in dogs.

## Teaser

This study identified biologically plausible metabolite- and pathway-level associations with fear-related behaviors in dogs, including touch sensitivity, dog-directed fear, combined fear, and separation-related behavior. These exploratory findings suggest that tryptophan, one-carbon/folate, and lipid metabolism may be relevant to fear and anxiety in dogs and could help guide future research on dietary and therapeutic strategies.

## Introduction

Fear, which is an adaptive response to a perceived threat, and anxiety, which is a response made in anticipation of a threat, are normal behavioral responses (Misslin 2003; Sherman and Mills 2008). Importantly, both fear and anxiety are closely interlinked with stress, as both real and perceived stressors trigger evolutionarily conserved responses (Chaouloff et al. 1999; Daviu et al. 2019). However, when behavioral and physiological responses to real or perceived threats become frequent, intense, or persist beyond the immediate context, they may compromise canine welfare and present challenges for owners (Tiira and Lohi 2014; Salonen et al. 2020). Indeed, behaviors commonly associated with fear or anxiety (eg destructiveness, excessive vocalization, house-soiling, and aggression) are among the most common behavioral concerns reported by dog caregivers and can strain the human–animal bond, reduce quality of life for dogs and people, and increase the likelihood of relinquishment to shelters (Patronek et al. 1996; New Jr et al. 2000; Bamberger and Houpt 2006; Fatjó et al. 2006). To evaluate fear and anxiety in dogs, validated behavioral assessment tools such as the Canine Behavioral Assessment and Research Questionnaire (C-BARQ) are widely used to identify behavioral phenotypes (Hsu and Serpell 2003) but also to link emotional states to physiological and genetic markers in canine research. These tools are valuable in exploring the multifactorial origins of fear-related behavior, which are shaped by a complex interplay among social and physical environments, genetics, and physiology (Beerda et al. 1999; Blackwell et al. 2013; Tiira and Lohi 2015; McGreevy et al. 2018; Edwards et al. 2019; Sarviaho et al. 2019; Starling et al. 2019).

Underlying these behavioral responses is a multifaceted interaction among internal systems, including the hypothalamic–pituitary–adrenal (HPA) axis, neuroendocrine and immunological signaling networks, and various metabolic pathways that collectively regulate stress responsiveness and emotional reactivity (Beerda et al. 1999; Ley et al. 2007). Among these regulatory frameworks is tryptophan (Trp) metabolism, which bridges nutritional input with neuroimmune function through two major downstream pathways: serotonin (5-HT) synthesis and kynurenine (KYN) degradation. Under conditions of chronic stress and immune activation, Trp metabolism can shift toward the KYN pathway through activation of Trp-catabolizing enzymes, including indoleamine 2,3-dioxygenase (IDO) (Gostner et al. 2020). This can shift Trp metabolism away from 5-HT synthesis and toward the KYN pathway, where some of the neuroactive KYN metabolites have been implicated in anxiety and affective disorders in humans (Arnone et al. 2018). Serotonin is a key modulator of fear and anxiety in mammals (Bacqué-Cazenave et al. 2020), although evidence directly linking the serotonergic system to behavior in dogs remains limited. Nevertheless, the effectiveness of serotonergic medications, particularly selective serotonin reuptake inhibitors, in reducing separation-related distress and certain forms of impulsive or fear-related aggression supports a role for serotonin in canine behavior (Simpson et al. 2007; Rosado et al. 2011). Yet studies examining peripheral concentrations of 5-HT or its metabolite, 5-hydroxyindoleacetic acid (5-HIAA), in dogs have reported inconsistent results. Some reported elevated concentrations in fearful or anxious dogs, while others described no differences (Riva et al. 2008; Alberghina et al. 2017; Riggio et al. 2020). These inconsistencies may suggest that fear/anxiety are not solely governed by a single metabolic pathway but rather emerge from complex interactions among multiple physiological systems.

Metabolomics has emerged as a promising tool to characterize physiology associated with behavior (Fearnley and Inouye 2016; Hagenbeek et al. 2016; Brennan et al. 2021). Previous studies comparing fearful and non-fearful, or anxious and non-anxious, dogs have identified differences in some metabolites between these groups using metabolomics. Puurunen et al. (2016b) reported alterations in oxidative stress markers, Trp-derived metabolites, and lipid profiles in fearful dogs, including indoxylsulfate and hypaphorine, both linked to Trp metabolism and decreased phospholipids. These findings may support the hypothesis that some forms of fearfulness may be associated with shifts in Trp metabolism, which could reduce Trp availability for 5-HT synthesis, potentially contributing to behavioral dysregulation. In a subsequent publication, Puurunen et al. (2018) found elevated plasma glutamine and γ-glutamyl glutamine in fearful dogs, suggesting effects of chronic psychological stress and oxidative imbalances. Additionally, Sacchettino et al. (2025) identified changes in metabolites related to γ-aminobutyric acid (GABA) and glutamate neurotransmission and bile acid metabolism in fearful dogs, further highlighting the involvement of multiple neurochemical systems. Additionally, there were significant changes in bile acid, lipid, and sphingolipid metabolism, which are linked to emotional and cognitive regulation.

Building on these insights, the present study expands on prior work through a larger sample size, inclusion of privately owned dogs from a broader range of breeds and breed types, and partial dietary control through provision of a standardized kibble prior to blood sample collection. This approach was intended to improve generalizability beyond single-breed populations while reducing diet-related variation in metabolomic profiles. The objectives of this study were to explore associations between specific owner-reported behavioral outcomes, including dog-directed fear, stranger- or human-directed fear, non-social fear, combined fear scores, touch sensitivity, separation-related behavior, and neuroticism, and peripheral physiological markers in a diverse population of pet dogs. Specifically, we investigated whether the behavioral categories derived from validated questionnaires (C-BARQ; Temesi et al. 2014) were associated with targeted serotonergic (5-HT, 5-HIAA, and the 5-HIAA:5-HT ratio) and untargeted serum metabolomic profiles. Together, these approaches provide a broader assessment of the physiological correlates of owner-reported fearfulness in dogs than could be achieved by examining serotonergic markers alone. In addition, we assessed the agreement of 5-HT and 5-HIAA rankings between enzyme-linked immunosorbent assay (ELISA) and untargeted metabolomics. A deeper understanding of these physiological relationships may inform targeted strategies to support fearful dogs. We hypothesize that dogs that have higher levels of owner-reported fearfulness show altered serotonergic activity (5-HT, 5-HIAA, and 5-HIAA:5-HT ratio) and metabolomic compounds in serum samples. Additionally, we hypothesize that 5-HT and 5-HIAA values obtained via ELISA and untargeted metabolomics will follow a similar rank order.

## Materials and methods

### Ethics statement

This study was conducted at the University of Guelph in accordance with national (Canadian Council of Animal Care) and institutional (Animal Use Protocol #5015) guidelines for the care and use of animals. Recruitment procedures and the collection of owner information were approved by the Research Ethics Board (REB #23-06-031).

### Animals, diets, and housing

One hundred healthy, adult dogs (48 females: 7 intact, 41 spayed; 52 males: 7 intact, 45 neutered) of various breeds (Table S1), with an average body weight of 25.7 ± 11.6 kg (mean ± SD), and average age of 4.2 ± 2.1 yr were used for this study. Dogs were recruited from 84 households in Guelph, ON, Canada and the surrounding areas using social media, email, and paper advertisements. Dogs were deemed healthy based on their previous medical history, complete blood count, and serum biochemistry panels and were not permitted to be receiving medication or supplements for a minimum of 4 wk prior to sample collection. All dogs were between 1 and 10 yr at the time of recruitment. Pregnant or intact females in estrus were excluded.

Dogs remained in their owners’ homes and continued to follow their normal daily routines during the diet acclimation period. Owners were provided with a commercial kibble (Acana Classics Red Meat Recipe, Champion Petfoods, Edmonton, AB, Canada; Table 1) to feed for at least 4 wk prior to researchers collecting a blood sample. Quantity and frequency of feeding were determined by owners. The diet was not intended as a treatment but was provided to standardize dietary nutrient profiles across subjects and reduce the number of potentially confounding factors, most importantly for metabolomics, as metabolites are affected by diet composition (Puurunen et al. 2022). Additionally, owners were asked to abstain from, or minimize, the use of treats for the week preceding the scheduled blood collection. Dogs were fasted prior to blood sample collection to minimize postprandial variation in circulating biomarkers.

**Table 1:** Guaranteed analysis of diet nutrient content of ACANA Red Meat Classics recipe on an as-fed basis and ingredient composition.^1^

Nutrient contents	Analyzed content (as-fed basis)
**Moisture (max, %)**	12
**Crude protein (min, %)**	27
**Crude fat (min, %)**	16
**Crude fiber (max, %)**	5
**Crude ash (max, %)**	8
**DHA (docosahexaenoic acid) (min, %)**	0.05
**EPA (eicosapentaenoic acid) (min, %)**	0.05
**Calcium (min, %)**	1.60
**Phosphorus (min, %)**	1.1
**Vitamin E (min, IU/kg)**	375
**Omega-3 fatty acids (min, %)**	0.3
**Omega-6 fatty acids (min, %)**	2.2
**Taurine (min, mg/kg)**	400
**Metabolizable energy, kcal/kg^2^**	3,425

1 Raw beef (16%), lamb meal (15%), pearled barley, whole peas, oat groats, pork fat (8%), pork meal (8%), whole oats, chickpea fiber, raw pork (2%), fish oil (1%), potassium chloride, dried kelp, salt, dried chicory root, fresh whole pumpkin, fresh whole butternut squash, fresh whole carrots, fresh whole apples, fresh whole pears, fresh whole zucchini, fresh kale, fresh spinach, fresh turnip greens, fresh beet greens, whole cranberries, whole blueberries, whole saskatoon berries, turmeric, milk thistle, burdock root, lavender, marshmallow root, and rosehips.

2 Calculated metabolizable energy based on modified Atwater values.

Owners received written and verbal instructions regarding all owner-completed study procedures, including feeding the standardized study diet, abstaining from or minimizing treat use during the week preceding blood collection, fasting their dog before the visit, and completing the behavioral surveys. Owners were asked to confirm that they had followed the relevant instructions. However, as the study involved client-owned dogs, adherence to these procedures could not be independently verified, and potential deviations should be considered when interpreting the results.

### Behavioral questionnaire

Dog owners were asked to fill out a survey about their dog’s behavior and usual daily routines. The survey was delivered online via Qualtrics (Seattle, WA, USA). Behavior-related questions were adapted from two previously published questionnaires: the Canine Behavioral Assessment and Research Questionnaire (C-BARQ; Hsu and Serpell 2003) and a temperament survey developed by Temesi et al. (2014). Questions were selected from both sources to capture variation in response scaling; C-BARQ items used intensity-based Likert-type scales for fear-related questions, while Temesi et al. (2014) used frequency-based Likert-type scales for similar behavior constructs. Items were named and grouped into behavioral categories determined from original publications (C-BARQ: dog-directed fear, stranger-directed fear, non-social fear, touch sensitivity, separation-related behavior; Temesi et al. 2014: dog-directed fear, human-directed fear, neuroticism). Separation-related questions from Temesi et al. (2014) were excluded due to overlap with frequency-based C-BARQ items for this category. Additionally, fear-related categories were combined for each survey to provide a combined fear category for each survey. All survey questions and their corresponding behavior categories used for analysis are presented in Table 2.

**Table 2:** Behavioral survey questions grouped by source questionnaire (Hsu and Serpell 2003; Temesi et al. 2014), including question prompts, response scales, and associated behavioral categories used for statistical analysis.

Temesi et al. (2014) Prompt: Please indicate how often your dog performs the following behaviors using the indicated scale. Scale: 0 = Never, 1 = Rarely, 2 = Sometimes, 3 = Usually, 4 = Always ^1^Questions are reverse scored These Likert-type items reflect frequency of behavior, and the combined fear category includes all questions from the neurocism, dog-directed fear, and human-directed fear categories.			
	Neuroticism		Your dog remains calm in tense situations^1^
			Your dog gets nervous easily
			Your dog adapts quickly to changes in its environment (eg being cared for by different people, moving house or a family member leaving home)^1^
			Your dog appears nervous and/or jumpy for several minutes after it has been startled
			Your dog appears calm in unfamiliar environments^1^
			Your dog appears calm in noisy, crowded places^1^
			Your dog has a specific fear or phobia
			Your dog acts anxious or fearful in response to sudden or loud noises
			Your dog acts anxious or fearful during thunderstorms
	Dog-directed fear		Your dog acts anxiously or fearfully when approached directly by an unfamiliar dog of the same or larger size
			Your dog behaves fearfully toward other dogs
			Your dog acts anxiously or fearfully when barked, growled, or lunged at by an unfamiliar dog
			Your dog acts anxiously or fearfully when approached directly by an unfamiliar dog of smaller size
			Your dog acts anxiously or fearfully when unfamiliar dogs visit your home
			Your dog behaves submissively (eg rolls over, avoids eye contact, licks lips) when greeting other dogs (fearfulness)
	Human-directed fear		Your dog acts anxiously or fearfully when an unfamiliar person tries to touch or pet the dog
			Your dog acts anxiously or fearfully when approached directly by an unfamiliar woman while away from home
			Your dog acts anxiously or fearfully when approached directly by an unfamiliar man while away from home
			Your dog acts anxiously or fearfully when approached directly by an unfamiliar child while away from home
			Your dog acts anxiously or fearfully when unfamiliar persons visit your home

C-BARQ (Hsu and Serpell 2003)Prompt (fear categories): Typical signs of mild to moderate fear include: avoiding eye contact, avoidance of the feared object, crouching or cringing with tail lowered or tucked between the legs, whimpering and whining, freezing, and shaking and trembling. Extreme fear is characterized by exaggerated cowering, and/or vigorous attempts to escape, retreat, defend themselves, or hide from the feared object, person, or situation. Please use the scale to indicate your own dog’s recent tendency to display fearful behavior in each of the following contexts:Scale (fear categories): 0 = No fear or anxiety (no visible signs of fear), 1–2 = Mild–moderate fear/anxiety, 3–4 = Extreme fear/anxiety (cowers, retreats, hides, etc.). These Likert-type items reflect intensity of behavior, and the combined fear category includes all questions from the stranger-directed fear, dog-directed fear, non-social fear, and touch sensitivity categories.			
	Stranger-directed fear		When approached directly by an unfamiliar adult while away from your home
			When approached directly by an unfamiliar child while away from your home
			When unfamiliar persons visit your home
			When an unfamiliar person tries to touch or pet the dog
	Dog-directed fear		When approached directly by an unfamiliar dog of the same or larger size
			When approached directly by an unfamiliar dog of smaller size
			When unfamiliar dogs visit your home
			When barked, growled, or lunged at by an unfamiliar dog
	Non-social fear		In response to sudden or loud noises (vacuum cleaner, car backfire, road drills, objects being dropped, etc.)
			In heavy traffic
			In response to strange or unfamiliar objects on or near the sidewalk (plastic trash bags, leaves, litter, flags flapping, etc.)
			During thunderstorms, firework displays, or similar events
			When first exposed to unfamiliar situations (first car trip, first time in elevator, first visit to veterinarian, etc.)
			In response to wind or wind-blown objects
	Touch sensitivity		When examined/treated by a veterinarian
			When having nails clipped by a household member
			When groomed or bathed by a household member
			When stepped over by a member of the household
			When having his/her feet toweled by a member of the household

C-BARQ (Hsu and Serpell 2003)Prompt (separation-related behavior): Some dogs show signs of anxiety or abnormal behavior when left alone, even for relatively short periods of time. Thinking back over the recent past, how often has your dog shown each of the following signs of separation-related behavior when left, or about to be left, on its own:Scale: 0 = Never, 1 = Seldom, 2 = Sometimes, 3 = Usually, 4 = AlwaysNote: These Likert-type items reflect frequency of behavior.			
**Separation-related behavior**		Shaking, shivering, or trembling	
		Excessive salivation	
		Restlessness, agitation, or pacing	
		Whining	
		Barking	
		Howling	
		Chewing or scratching at doors, floor, windows, curtains, etc.	
		Loss of appetite	

### Blood collection

Dogs were fasted for a minimum of 10 h prior to the collection of a single blood sample. For most dogs, blood was collected via jugular venipuncture using a 20- or 21-gauge needle and a 10 mL syringe. However, in response to the dog’s level of comfort with different positions or methods of restraint, cephalic or saphenous venipuncture was used in instances where they reduced observable signs of distress in the dog. In order to minimize discomfort from the needle, Emla cream (lidocaine 2.5% and prilocaine 2.5%; Aspen Pharmacare Canada, Oakville, ON, Canada) was applied to the collection area and allowed to sit before blood was collected. Of the collected blood, approximately 7 mL was put into a serum vacutainer (Becton, Dickinson and Company, Franklin Lakes, NJ, USA) and allowed to clot. Blood was centrifuged at 2,500 rpm for 15 min using an IEC Centra CL2 (Thermo Electron Corp., Milford, MA, USA), then the serum aliquots were collected and frozen at −80 °C until later analysis.

### Serum 5-HT and 5-HIAA analysis

Commercially available ELISA kits were used to analyze serum 5-HT (Canine Serotonin/5-Hydroxytryptamine ELISA Kit, MBS738294, MyBioSource Inc., San Diego, CA, USA) and its derivative, 5-HIAA (Canine 5-Hydroxyindoleacetic acid ELISA MBS745172, MyBioSource Inc., San Diego, CA, USA). All samples (*n *= 100) were assayed undiluted in duplicate in accordance with the manufacturer’s instructions, with a CV < 15% considered acceptable. Samples below the assay’s lower limit of quantification were re-assayed; values that remained below the quantifiable range were considered not quantifiable and treated as missing. The 5-HIAA:5-HT ratio was calculated using the resulting concentrations from the ELISA analysis. Final sample sizes were *n = *97 for 5-HT, *n *= 99 for 5-HIAA, and *n *= 96 for the 5-HIAA:5-HT ratio.

### Serum metabolomics analysis

Serum samples (*n *= 100) were shipped on dry ice from −80 °C storage to The Metabolomics Innovation Center (TMIC), University of Alberta (Edmonton, AB, Canada) for analysis using a chemical isotope labeling LC–MS platform (Elevated Global Metabolomics), which provides broad untargeted metabolome coverage with semi-targeted relative quantification of detected metabolites. Samples were randomized prior to processing. A pooled reference sample was prepared by combining 20 μL from each individual sample. Individual samples were vortexed and centrifuged at 15,000 × *g* for 1 min, after which the supernatant was aliquoted into four portions for different labeling strategies, backup, and pooled sample preparation. Protein precipitation was performed by adding 90 μL of LC–MS grade methanol, followed by incubation at −20 °C for 30 min. Extracts were dried and temporarily stored at −80 °C until labeling.

Chemical isotope labeling was performed using Dansyl-labeling kits (for amine/phenol groups) and DmPA-labeling kits (for carboxyl groups; NovaMT Inc., Edmonton, AB, Canada) following the manufacturer’s protocols. The isotope labeling strategy increases the mass and ionization efficiency of small metabolites, allowing them to be reliably detected within the 220–1,000 m/z scan window and quantified based on accurately paired 12C/13C signal (Zhao et al. 2016). For amine/phenol labeling, 25 μL LC–MS grade water, 12.5 μL buffer reagent, and 37.5 μL of either 12C2- or 13C2-labeled reagent were added to each sample. Samples were vortexed, centrifuged, and incubated at 40 °C for 45 min. A 7.5 μL quenching reagent was added, followed by a 10-min incubation and addition of 30 μL of pH-adjusting reagent. For carboxyl labeling, 25 μL acetonitrile/water (3:1, v/v), 10 μL catalyzing reagent, and 25 μL of labeled reagent were added, followed by incubation at 80 °C for 60 min. After adding 40 μL quenching reagent, samples were incubated for another 30 min to complete labeling. After labeling, each 12C-labeled individual sample was mixed 1:1 (v/v) with the 13C-labeled pooled reference for relative quantification; quality control samples were prepared as a 1:1 mix of 12C- and 13C-labeled pooled samples.

Labeled samples were analyzed using a Thermo Vanquish LC system coupled to a Bruker Impact II QTOF mass spectrometer with a reversed-phase C18 column. Chromatographic separation was achieved using a formic acid–water/acetonitrile gradient at a flow rate of 400 μL/min and column temperature of 40 °C. Mass spectra were acquired over an m/z range of 220–1,000 at 1 Hz. Quality control samples were injected every 20 runs to monitor instrument performance.

Data quality was verified using consistent background peaks (*m*/*z* 251.0849 for amine/phenol and *m*/*z* 242.0175 for carboxyl channels), confirming mass accuracy across all runs. Retention time alignment was validated using seven calibrant peaks per channel. A total of 214 LC–MS runs (including six quality control samples per channel) were exported as .csv files using Bruker DataAnalysis 4.4 and processed in IsoMS Pro 1.3.0. Peak extraction was performed with a retention time tolerance of 9 s and mass tolerance of 10 ppm. To reduce noise, metabolites were retained only if present in at least 80% of samples (Bijlsma et al. 2006). Data were normalized using the ratio of total useful signal.

Metabolite identification followed a three-tier strategy implemented in IsoMS Pro 1.3.0 (NovaMT Inc.) using the NovaMT Metabolite Database v3.0. In Tier 1, peak pairs were searched against the labeled metabolite library (CIL Library) based on accurate mass (±10 ppm) and retention time (±10 s). The CIL Library contains >1,500 experimental entries and, therefore, Tier 1 provides the highest-confidence identifications. In Tier 2, remaining peak pairs were searched against the linked identity library (LI Library), which includes >9,000 pathway-related metabolites and provides high-confidence putative identifications based on accurate mass and predicted retention time matching. In Tier 3, remaining peak pairs were matched by accurate mass only to the MyCompoundID (MCID) library, which is composed of known endogenous metabolites (zero-reaction library) and predicted products from one- and two-step metabolic reactions (one- and two-reaction libraries). Only Tier 1 and Tier 2 metabolites with identified KEGG IDs were included in statistical analysis. Because Tier-3 identification is based on mass-only matching, the exact structure can be difficult to determine (eg isomers/isobars); therefore, Tier-3 annotations were not used for inferential statistics in this study.

### Statistical analysis

All statistical analyses were performed in SAS Studio (Version 9.4, SAS Institute Inc., Cary, NC, USA). Ordinal logistic regression models using the GLIMMIX procedure were used to evaluate the relationship between the frequency of responses for each dog in each of the 10 behavioral categories and each metabolite identified through metabolomics, and 5-HT, 5-HIAA, and the 5-HIAA:5-HT ratio. Models were fit with a multinomial distribution and cumulative logit link with dog included as a random effect. Sex and age were included as covariates, as these factors have been identified as affecting metabolite profiles (Puurunen et al. 2022). Breed was not included due to the diverse study population, the large proportion of mixed-breed dogs, and the small number of dogs represented within most individual breed categories. Body weight was included as a covariate in place of breed to account, in part, for size-related variation. Body weight was included for all behavioral categories except combined fear (C-BARQ) and combined fear (Temesi et al. 2014), as inclusion of body weight in these models resulted in model instability and non-convergence. For 5-HT, 5-HIAA, and the 5-HIAA:5-HT ratio, significance was determined at a *P* ≤ 0.05 and a trend at *P* ≤ 0.10.

At the metabolite level for metabolomics data, raw *P*-values were corrected for multiple hypothesis testing using Benjamini–Hochberg false discovery rate (FDR) within each behavioral category, and metabolites were considered significant at FDR-adjusted *P* (*P*_FDR_) ≤ 0.05. To complement these analyses, pathway analysis was performed in MetaboAnalyst (version 6.0) using metabolites with raw *P* ≤ 0.05 as input. This approach was used to assess whether groups of metabolites showing nominal associations clustered within biological pathways, which might indicate biologically meaningful patterns even if individual metabolites were not significant after FDR. Pathway significance was determined by FDR-corrected hypergeometric tests with relative-betweenness centrality, using all 580 KEGG-identified metabolites in our dataset as the reference metabolome and the KEGG *Canis lupus familiaris* pathway library. Additionally, Spearman’s rank correlation using the CORR procedure was used to assess associations between ELISA- and metabolomics-derived values for serotonin (5-HT) and 5-HIAA.

## Results

The frequency distribution of survey item responses by behavioral category is provided in Table S2.

### Quantitative analysis of serum 5-HT, 5-HIAA, and 5-HIAA:5-HT ratio

All statistical results, including odds ratios and 95% confidence intervals, are summarized in Table 3. A higher 5-HIAA:5-HT ratio was associated with lower separation-related behavior scores (1.15 [1.02–1.29]; odds ratio estimate [95% CI]; *P* = 0.03), suggesting that dogs with more 5-HT turnover experienced less separation anxiety. No other survey categories were associated with the 5-HIAA:5-HT ratio (*P* > 0.05). There was a positive association between 5-HIAA and combined fear score (Temesi et al. 2014; 0.9943 [0.9889–0.9998]; *P* = 0.04), and combined fear (C-BARQ) also tended to be positively associated with 5-HIAA (0.9942 [0.9899–1.0000]; *P* = 0.08). No other survey categories were associated with 5-HIAA (*P* > 0.10). There were no associations between 5-HT and any survey categories (*P* > 0.10).

**Table 3:** Odds ratio estimates^1^ and 95% confidence intervals for associations between 5-HT (*n *= 97), 5-HIAA (*n *= 99), and the 5-HIAA:5-HT ratio (*n *= 96) and owner-reported behavioral outcomes for healthy, adult dogs of various breeds.

Surveycategory		5-HT	5-HIAA	5-HIAA:5-HT ratio
**C-BARQ**	Dog-directed fear	1.00 (0.99–1.01)	0.99 (0.98–1.01)	1.13 (0.90–1.42)
	Stranger-directed fear	1.00 (0.99–1.01)	0.99 (0.97–1.01)	1.14 (0.81–1.60)
	Non-social fear	1.00 (0.99–1.00)	1.00 (0.99–1.00)	1.05 (0.89–1.24)
	Touch sensitivity	1.00 (1.00–1.00)	1.00 (0.99–1.00)	1.10 (0.97–1.26)
	Combined fear	1.00 (1.00–1.00)	0.99 (0.99–1.00)*	1.01 (0.91–1.14)
	Separation-related behavior	1.00 (1.00–1.00)	1.00 (0.99–1.01)	1.15 (1.02–1.29)**
**Temesi et al. (2014)**	Dog-directed fear	1.00 (0.99–1.00)	1.00 (0.99–1.00)	1.01 (0.87–1.17)
	Human-directed fear	1.00 (0.99–1.01)	0.99 (0.97–1.00)	1.04 (0.75–1.44)
	Neuroticism	1.00 (1.00–1.00)	1.00 (0.99–1.00)	1.03 (0.91–1.16)
	Combined fear	1.00 (1.00–1.00)	0.99 (0.99–1.00)**	0.97 (0.87–1.08)

1 Models the probability of selecting lower ordinal responses (ie reduced fearfulness) in association with each biomarker. As such, an OR <1 indicates greater fear or sensitivity, while an OR >1 suggests lower fear.

** *P* < 0.05.

* *P* < 0.10.

### Metabolomic feature annotation and filtering

Following filtering and normalization, an average of 6,178 ± 29 peak pairs per sample were retained across both channels. Of the 6,284 unique peak pairs detected, 5,490 (87.4%) were either positively identified or putatively matched. High-confidence identifications (Tiers 1 and 2) accounted for 1,266 peak pairs. Specifically, 430 peak pairs were positively identified via the CIL Library, and 836 were matched in the LI Library. The MCID library yielded 1,221, 2,251, and 752 matches in the zero-, one-, and two-reaction tiers, respectively. For downstream statistical analyses, we restricted the dataset to Tier 1 and Tier 2 metabolites with assigned KEGG IDs, removed duplicate annotations arising from isomer identifications, and applied an 80% presence threshold (retaining metabolites detected in ≥80% of samples), resulting in 580 metabolites included in the final analysis.

### Metabolite-level associations

Following FDR correction, no metabolites were associated with any survey categories from Temesi et al. (2014) (neuroticism, dog-directed fear, human-directed fear, or combined; *P* > 0.05). For the C-BARQ categories, non-social fear, stranger-directed fear, and separation-related behavior were not associated with any metabolites (*P* > 0.05). Lauric acid (KEGG ID: C02679), a medium-chain saturated fatty acid involved in lipid metabolism and β-oxidation pathways, was positively associated with touch sensitivity (0.63 [0.51–0.78]; *P*_FDR_ = 0.007; reviewed in Dayrit 2015). Dog-directed fear (C-BARQ) was positively associated with both 7,8-dihydrobiopterin (BH2; KEGG ID: C02953; 0.0004 [0.00–0.04]; *P*_FDR_ = 0.004) and tetrahydrobiopterin (BH4; KEGG ID: C00272; 0.003 [0.0001–0.0685]; *P*_FDR_ = 0.004), which functions as a cofactor for tryptophan hydroxylase. Odds ratio estimates for BH₂ and BH₄ were very small with unstable confidence intervals, and as such, these associations should be interpreted with caution. Finally, formiminoglutamic acid (FIGLU; KEGG ID: C00439), an intermediate of histidine catabolism linked to folate metabolism, was positively associated with combined C-BARQ fear scores (0.11 [0.04–0.28]; *P*_FDR_ = 0.004) (reviewed in Brosnan and Brosnan 2020).

Based on raw *P*-values, various metabolites were significantly associated with behavioral categories. These data are summarized in Table 4 but should be interpreted as exploratory only.

**Table 4:** Number of metabolites^1^ with raw (unadjusted) *P* < 0.05 for each behavioral category, organized by pathway.

Pathway	C-BARQ						Temesi et al. (2014)			
	Dog-directed fear	Stranger-directed fear	Non-social fear	Touch sensitivity	Combined fear	Separation-related behavior	Dog-directed fear	Human-directed fear	Neuroticism	Combined fear
**Histidine metabolism**	1	3	1	4	4	0	3	2	4	4
**Tryptophan metabolism**	2	11	2	17	11	5	7	9	11	11
**Lysine metabolism**	2	2	5	3	4	1	6	0	3	3
**Arginine and proline metabolism**	2	3	2	2	4	0	5	2	3	3
**Phenylalanine metabolism**	2	3	2	1	3	0	3	3	3	3
**Folate biosynthesis**	3	1	0	1	4	1	4	0	3	3
**Pyrimidine metabolism**	3	0	1	1	1	0	4	0	1	1
**Cysteine and methionine metabolism**	0	0	1	0	0	0	0	0	2	2
**Purine metabolism**	1	0	1	0	0	0	1	1	1	1
**Valine, leucine, isoleucine metabolism**	1	3	0	1	2	0	2	1	2	2
**Tyrosine metabolism**	2	4	0	1	1	1	3	2	3	3
**Fatty acid metabolism**	2	0	2	3	1	1	1	1	1	1
**Other/pathway not identified^2^**	17	19	20	24	27	14	26	13	24	24
**Total**	38	49	37	58	62	23	65	34	61	61

^1^Counts from ordinal logistic regression models and adjusted for age, body weight, and sex in privately owned dogs of various breeds (*n *= 100).

2 Summarizing metabolites that could not be assigned to a KEGG pathway, metabolites from pathways represented by fewer than two associated metabolites, or metabolites involved in multiple pathways that were not assigned to a single pathway category.

### Pathway analysis

**Figure 1:**
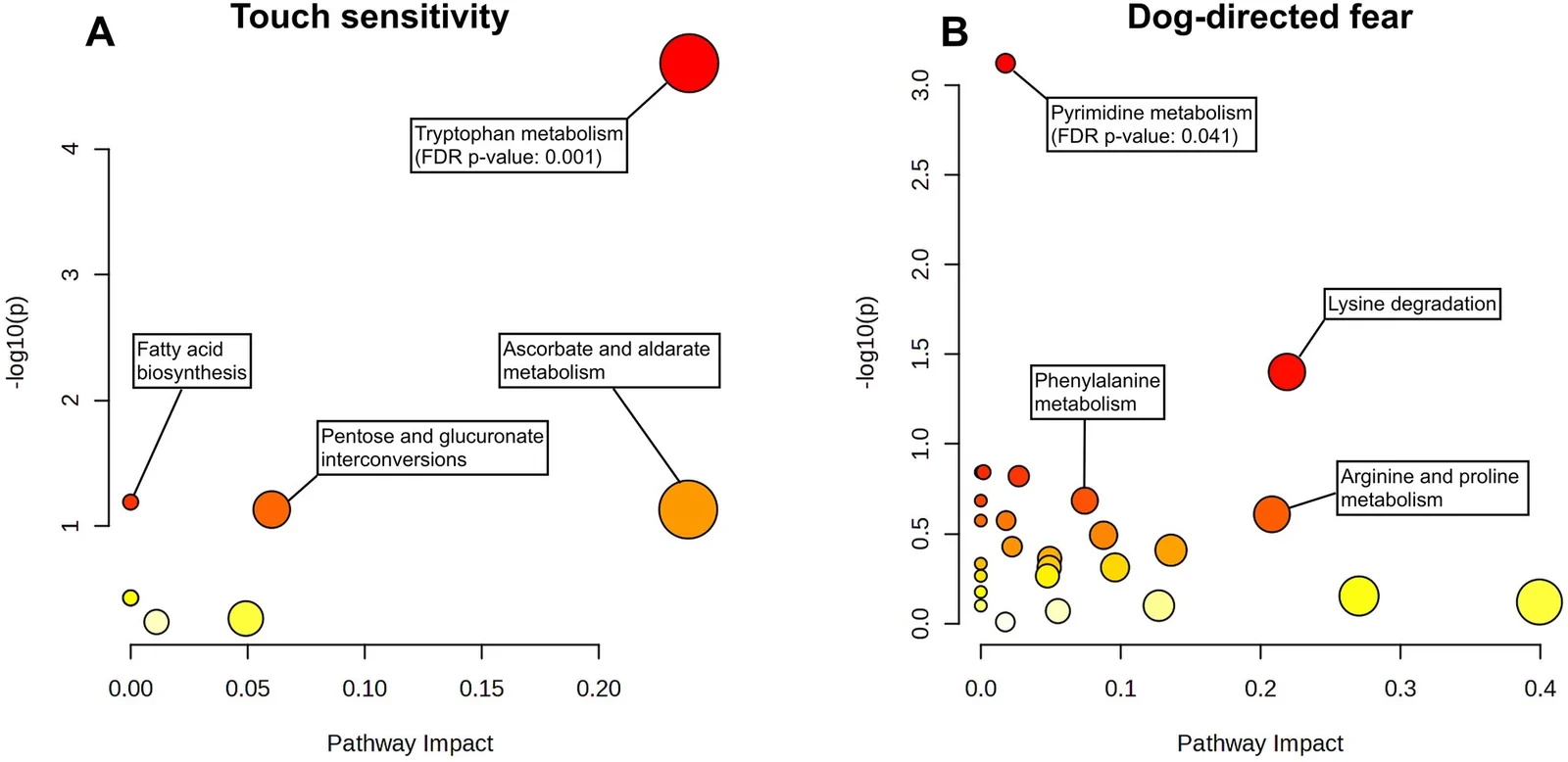
Bubble plots of pathway enrichment/topology for touch sensitivity (A) and dog-directed fear (B) (Temesi et al. 2014). Input metabolites were those with raw *P* < 0.05 in ordinal logistic models adjusting for age, body weight, and sex; x-axis: pathway impact, y-axis: −log10(P). Bubble size scales with impact; color reflects nominal P. Only tryptophan metabolism (B; FDR *P* = 0.001) and pyrimidine metabolism (A; FDR *P* = 0.041) were significant after FDR; others are shown for context (A FDR = 0.997; B FDR = 1.000). Pathways were mapped using KEGG in MetaboAnalyst 6.0.

**Figure 2:**
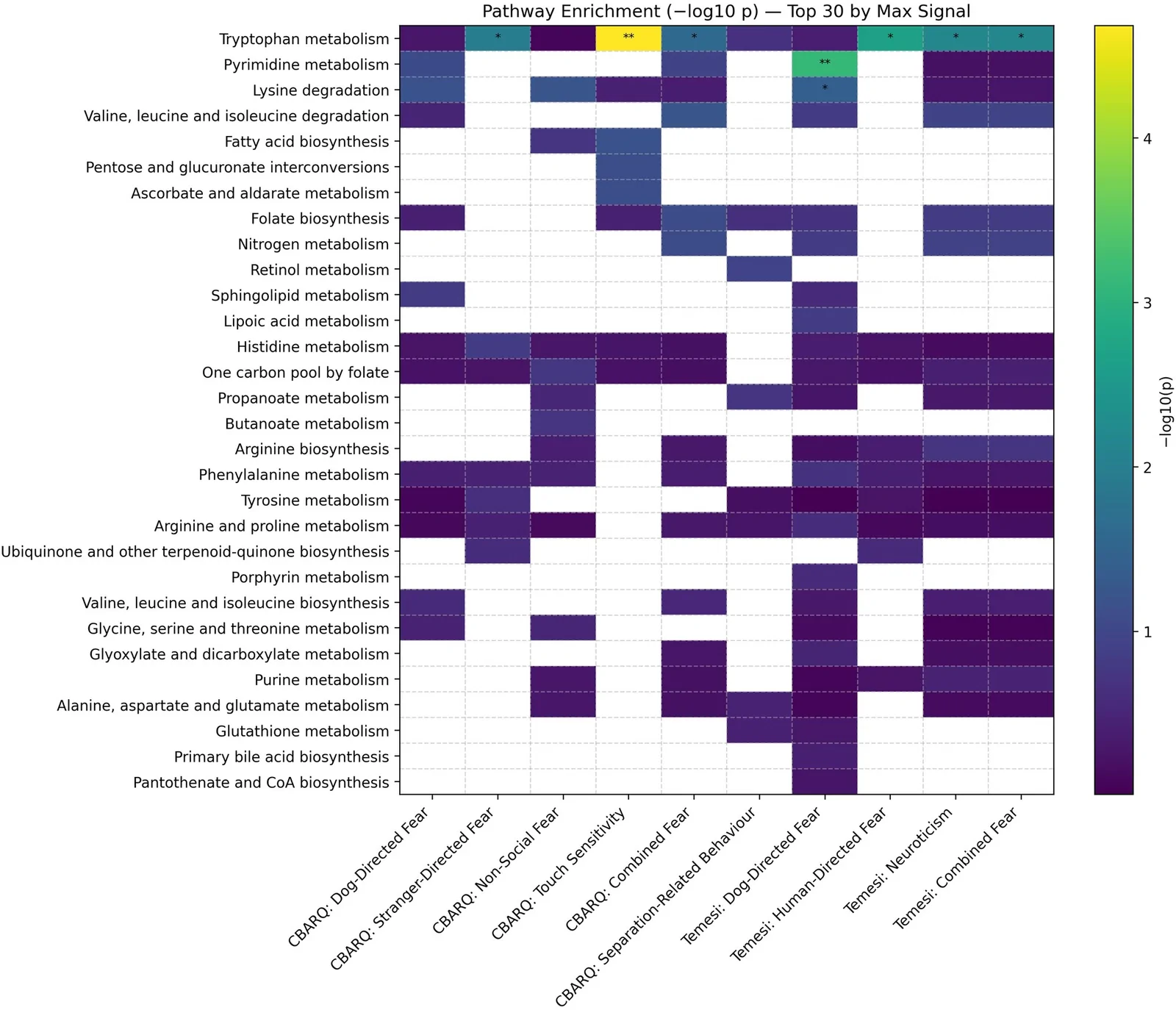
Heatmap of pathway enrichment across behavioral categories. Cells show − log10(P) from KEGG pathway enrichment/topology (MetaboAnalyst 6.0); rows are pathways (top 30 by maximum signal) and columns are behavioral categories. Asterisks mark significance (*unadjusted *P* < 0.05; **FDR < 0.05). Input metabolites were those with raw *P* < 0.05 in ordinal logistic models adjusted for age, body weight, and sex (*n *= 100 dogs).

The Trp metabolism pathway was significantly enriched for six behavior categories, though only the touch sensitivity behavioral category met FDR correction (Figures 1 and 2). Specifically, 8 of the 22 identified metabolites within the Trp pathway were associated with touch sensitivity (raw *P* < 0.05), resulting in pathway-level *P*_FDR_ = 0.001 (Impact: 0.239; Figure 1).

For dog-directed fear (Temesi et al. 2014), the pyrimidine metabolism pathway was enriched, with five of the seven identified metabolites from the pathway having a raw *P* < 0.05 (Pathway *P*_FDR_ = 0.04; Impact = 0.018; Figure 1). Dog-directed fear (Temesi et al. 2014) was also enriched for lysine degradation, though it did not meet FDR correction (Figure 2). No additional pathways were significantly enriched for any other behavioral categories, based on either raw *P*-values or pathway-level FDR–corrected *P*-values.

### Comparison of ELISA and metabolomics analysis

**Figure 3:**
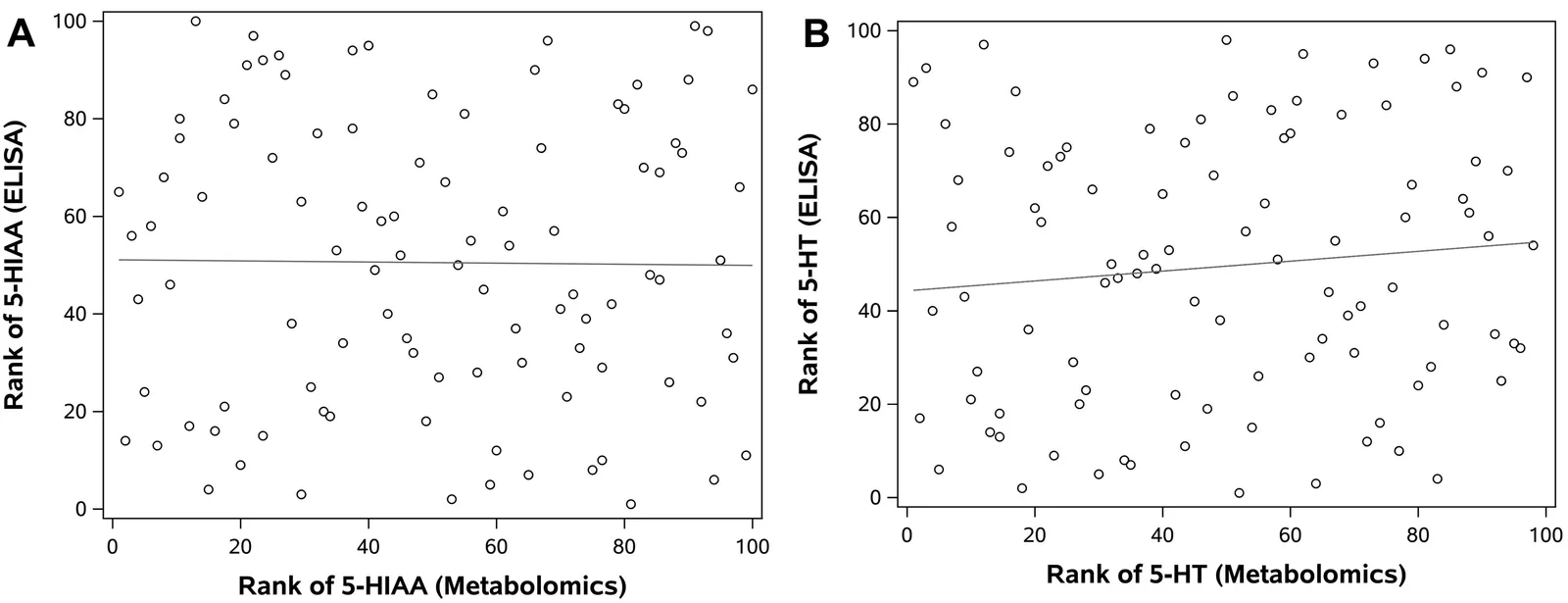
Spearman correlation plots comparing ELISA and metabolomics-derived rank values for 5-HIAA (A) and serotonin (5-HT) (B).

There was no correlation between ELISA and metabolomics-derived values for serotonin (Spearman’s ρ  =  0.106, *P* = 0.30) or 5-HIAA (ρ = –0.01, *P* = 0.91) (Figure 3).

## Discussion

Findings from the current study suggest that Trp metabolism, along with markers related to oxidative stress, lipid metabolism, and folate-dependent metabolism/formiminoglutamic acid, may play a role in modulating fear-related behavior in dogs. In companion animals and other species, fear- or anxiety-related behaviors are common, but the molecular underpinnings are still not well defined. The present study aimed to explore physiological correlates of fearfulness in dogs using both targeted ELISA-based analysis of serotonergic markers and LC–MS metabolomics. Importantly, behavior was assessed using the C-BARQ and the temperament survey by Temesi et al. (2014), which allowed for standardized quantification of fear-related behaviors across different categories. Although targeted analysis of 5-HT and 5-HIAA did not show consistent associations across all behavioral categories, the broader metabolomics results identified Trp metabolism as the most consistent pathway-level signal, supporting further consideration of this pathway in relation to canine fearfulness.

For the targeted serotonergic markers, we observed no associations between 5-HT and any behavioral category, suggesting that circulating 5-HT may not be a consistent marker of fearfulness across populations and behavioral assessment approaches. However, 5-HIAA showed a significant but small positive association with combined fear (Temesi et al. 2014) and tended to be positively associated with combined fear (C-BARQ), while the 5-HIAA:5-HT ratio was negatively related to separation-related behavior. These findings suggest that 5-HIAA and serotonin turnover may provide additional information beyond 5-HT alone, although the small effect sizes and limited consistency across behavioral categories indicate that these results should be interpreted cautiously.

Previous studies examining targeted Trp-related markers in dogs have also reported mixed findings. A study comparing anxious (*n *= 20) and non-anxious (*n *= 13) dogs found higher concentrations of 5-HT and 5-HIAA in plasma of anxious dogs (Riva et al. 2008). In contrast, a shelter-dog study (*n *= 14) identified a weak relationship between 5-HT concentrations and human-directed fearfulness where dogs with lower serum 5-HT levels were scored as being more fearful in a human interaction test (Alberghina et al. 2017), and another shelter-dog study (*n *= 39) found no differences in Trp or 5-HT based on behavioral presentation (Riggio et al. 2020). Together, these results suggest that peripheral serotonergic markers may relate to fear- or anxiety-related behavior in some contexts, but are likely influenced by differences in study population, sample type, and behavioral assessment approach.

While the targeted serotonergic findings were not consistent across all behavioral categories, the LC–MS metabolomics results provided broader support for Trp metabolism as a pathway of interest. Trp metabolism was enriched for touch sensitivity scores and showed nominal associations across 6 of 10 behavioral categories. Additionally, Trp metabolism showed consistent associations across multiple behavioral categories, including touch sensitivity (17 metabolites), combined fear (11 metabolites), and neuroticism (11 metabolites), supporting its relevance to fear-related behavior. Tryptophan is an indispensable amino acid for dogs, serving as a substrate for protein synthesis and as a precursor to the KYN and 5-HT pathways (Ruddick et al. 2006), with both implicated in emotional regulation (Arnone et al. 2018; Bacqué-Cazenave et al. 2020). Chronic stress and immune activation can shift Trp metabolism toward the KYN pathway via IDO activation, potentially reducing 5-HT synthesis and contributing to emotional and behavioral changes/dysregulation (O’Mahony et al. 2015). Beyond this, there were positive associations between dog-directed fear (C-BARQ) and BH4 and its oxidized form, BH2. BH4 is a cofactor for Trp hydroxylase, the rate-limiting enzyme in 5-HT synthesis (Gostner et al. 2020). An elevated BH2: BH4 ratio is a marker of oxidative stress and reflects reduced BH4 availability, which may impair 5-HT production and may contribute to altered emotional reactivity. Although the corresponding effect estimates were imprecise, their direction and biological role support a cautious interpretation in the context of the broader Trp pattern and warrant further investigation. Together, these findings suggest that peripheral 5-HT alone may not fully capture fear-related behavior in dogs, but that broader patterns within Trp metabolism may still provide useful insight into the biological processes associated with fearfulness.

Of the three previous studies that have used metabolomics to investigate metabolic differences between fearful and non-fearful dogs, one study reported differences in compounds related to Trp metabolism, as well as markers of oxidative stress and lipid metabolism. All dogs included in the study were Great Danes (*n* = 10 fearful, 10 non-fearful), though the study did not control for diet (Puurunen et al. 2016b). A diet-controlled follow-up in both Great Danes and German Shepherds (*n* = 20 fearful, 21 non-fearful) did not find differences in metabolites related to Trp metabolism (Puurunen et al. 2018), nor did a study of privately owned dogs of mixed breeds without diet control (*n* = 8 fearful, 8 non-fearful) (Sacchettino et al. 2025). Separately, plasma metabolomics in German Shepherds with ADHD-like scores (*n* = 22) identified Trp-related metabolites and lipid metabolism differences (Puurunen et al. 2016a). Taken together, these studies, alongside the results from the present study, suggest that Trp metabolism may be relevant for fear-related behavior in dogs, but findings may vary across populations, diets, breeds, and behavioral assessment methods.

The use of both targeted and untargeted analyses is unique to this study with the hope of offering complementary insights into the benefits of using both methods. Specifically, our study is the first to compare ELISA-derived 5-HT and 5-HIAA in dogs and metabolomics-derived rank values. Interestingly, no correlation was observed between ELISA‑ and metabolomics‑derived values. This discrepancy likely reflects methodological differences. The LC–MS method in this study used spectra over an *m*/*z* range of 220–1,000 and used carbon labeling to increase the mass of lower-mass molecules, such as 5-HT and 5-HIAA, so they fell within this range (Zhao et al. 2016). In contrast, ELISA provides quantification of the molecules. These findings underscore the importance of both targeted and untargeted approaches in biomarker research. Although this comparison covers only two metabolites among many profiled in metabolomics and likely reflects platform differences rather than a biological explanation, the lack of correlation underscores the need for further work directly comparing platforms and supports using both targeted and untargeted approaches in future studies to improve the accuracy and reproducibility of biomarker discovery.

In addition to Trp metabolism, lauric acid was positively associated with touch sensitivity scores. Lauric acid is a medium-chain saturated fatty acid involved in lipid metabolism and β-oxidation pathways (reviewed in Dayrit 2015), which is consistent with findings from previous studies in both dogs and other species (Puurunen et al. 2016b; Meeder et al. 2021; Padilha et al. 2024). Differences in lipid metabolism have also been identified in relation to other behavioral phenotypes, including ADHD-like behavior in dogs and mood disorders in humans (Puurunen et al. 2016a; Meeder et al. 2021). Lipid metabolism may influence neuroinflammation and oxidative stress, both of which can modulate emotional reactivity.

Pyrimidine metabolism was enriched for dog-directed fear, while no other pathways or individual pyrimidine metabolites were associated with behavioral categories. Pyrimidine metabolism encompasses the synthesis, degradation, and salvage of pyrimidine bases (cytosine, thymine, uracil), which are essential for DNA, RNA, and other cellular molecules like lipids and may relate to anxiety or fear through indirect mechanisms. De novo pyrimidine synthesis is coupled to mitochondrial respiration, so shifts in this pathway can mirror cellular energy and redox states that influence neural function (Fang et al. 2013). Extracellular pyrimidine nucleotides can also signal via neuronal and astrocytic receptors, modulating glutamate and GABA signaling and potentially altering excitatory to inhibitory tone, both critical for emotional regulation (Guzman and Gerevich 2016).

Formiminoglutamic acid (FIGLU) is an intermediate of histidine catabolism (Cooperman and Lopez 2002). Elevated FIGLU is a marker of impaired folate-dependent one-carbon metabolism, which is important for nucleotide synthesis and for maintaining methyl-group availability for DNA methylation and other methylation reactions, including those involved in neurotransmitter pathways (Crider et al. 2012). Disturbances in folate and one-carbon status have been associated with depression, and in some studies with anxiety, in humans (Bjelland et al. 2003; Gilbody et al. 2007). In the current study, the positive association between FIGLU and combined C-BARQ fear scores could indicate the contribution of one-carbon and methylation-related processes to variation in fearfulness in dogs.

This study demonstrates the benefits of using a metabolomics approach to investigate fear-related behavior in dogs, though we recognize the technical and theoretical limitations that should be addressed. A major consideration in interpreting our findings and in comparing them to other work in dogs is that there is no standardized approach to defining or measuring fear- or anxiety-related behavior. Prior studies have used laboratory-developed surveys or behaviorist classifications and often group dogs into binary groups (eg fearful vs. non-fearful). That approach is statistically convenient, but it treats “fearful” as if it were a single, uniform state and assumes that dogs classified as fearful in one study are directly comparable to those classified as fearful in another. In the present study, we did not impose a binary classification. Instead, we modeled owner-reported scores for specific behavior categories using two published questionnaires. The C-BARQ-derived categories emphasize perceived intensity of a behavior, whereas Temesi et al. (2014) focuses on the frequency of a behavior in a defined scenario. Taken together, the lack of standardized behavioral tests and the use of different grouping strategies across studies likely contribute to differences in which metabolites and pathways appear to be associated with fear-related behavior. Future studies should consider our approach and a wider range of fearfulness categories. These findings identify tryptophan metabolism, lipid metabolism, and folate-dependent one-carbon metabolism as promising targets for the development of nutritional strategies to reduce fearfulness in dogs. Although specific feeding recommendations cannot yet be made, these results provide a biological basis for future controlled feeding studies designed to determine which dietary interventions can beneficially modify these pathways and improve fear- and anxiety-related outcomes.

## Conclusion

This study highlights the potential of combining untargeted metabolomics with targeted measures to explore physiological markers associated with fear-related behaviors in privately owned dogs. Tryptophan metabolism was the pathway most consistently associated with fear-related behaviors, with additional findings involving one-carbon, nucleotide, and lipid metabolism. However, associations varied across behavioral outcomes, and few remained significant following FDR correction. Future research should aim to validate these findings in a longitudinal study and explore targeted nutritional interventions to support canine well-being and to test causal links between diet, metabolism, and behavior.

## Supplementary Material

skag260_Supplementary_Data

## Abbreviations

5-HIAA: 5-hydroxyindoleacetic acid
5-HT: serotonin
BH2: 7,8-dihydrobiopterin
BH4: tetrahydrobiopterin
C-BARQ: Canine Behavioral Assessment and Research Questionnaire
ELISA: enzyme-linked immunosorbent assay
FDR: false discovery rate
FIGLU: formiminoglutamic acid
GABA: γ-aminobutyric acid
HPA: hypothalamic–pituitary–adrenal
IDO: indoleamine 2,3-dioxygenase
KYN: kynurenine
Trp: tryptophan
