# Supplementary material for: Investigating targeted and untargeted approaches to biomarker discovery in fearful, adult dogs
Source: J Anim Sci. 2026 Aug 19;104:skag260. doi: 10.1093/jas/skag260 (PMC13552375; doi:10.1093/jas/skag260)
Supplement: Supplementary file 1 [file skag260_supplementary_data.docx]

| **Breed** | **Number of Dogs** |
| --- | --- |
| Mixed breed | 46 |
| Designer breed^2^ | 13 |
| Purebred | 41 |
| Golden Retriever | 5 |
| Labrador Retriever | 4 |
| Australian Shepherd | 3 |
| Czechoslovakian Wolfdog | 3 |
| Dachshund | 3 |
| German Shepherd | 3 |
| English Springer Spaniel | 2 |
| German Shorthaired Pointer | 2 |
| Husky | 2 |
| Border Collie | 1 |
| Border Terrier | 1 |
| Corgi | 1 |
| Great Dane | 1 |
| Havanese | 1 |
| Irish Terrier | 1 |
| Miniature Poodle | 1 |
| Rottweiler | 1 |
| Samoyed | 1 |
| Shetland Sheepdog | 1 |
| Standard Poodle | 1 |
| Tibetan Terrier | 1 |
| Vizsla | 1 |
| Yorkshire Terrier | 1 |

**Supplementary Table S1.** Breed^1^ distribution of privately owned dogs included in the study.

^1^ Breed classification was owner-reported.

^2^Designer breeds refer to intentional crosses between two recognized dog breeds (e.g., Labradoodle, Puggle, etc.)

**Supplementary Table S2.** Frequency distribution of survey item responses by behavioural category^1^.

| Survey Category | | Frequency of Answer | | | | |
| --- | --- | --- | --- | --- | --- | --- |
|  |  | 0 | 1 | 2 | 3 | 4 |
| CBARQ | dog-directed fear | 116 | 83 | 87 | 44 | 42 |
|  | stranger-directed fear | 114 | 74 | 92 | 57 | 61 |
|  | non-social fear | 206 | 138 | 142 | 52 | 55 |
|  | touch sensitivity | 212 | 97 | 99 | 40 | 44 |
|  | combined fear | 648 | 392 | 420 | 193 | 202 |
|  | separation-related behavior | 488 | 132 | 104 | 43 | 21 |
| Temesi et al., 2014 | dog-directed fear | 147 | 153 | 131 | 62 | 84 |
|  | human-directed fear | 116 | 95 | 101 | 96 | 90 |
|  | neuroticism | 142 | 192 | 216 | 218 | 124 |
|  | combined fear | 405 | 440 | 448 | 376 | 298 |

^1^ Values represent the number of responses for each score option across all dogs, summarized within each behavioural category. Missing responses were excluded from the frequency counts; total missing responses = 270.
